# Literature review and expert opinion on the treatment of high-risk acute myeloid leukemia in patients who are eligible for intensive chemotherapy

**DOI:** 10.3389/fonc.2024.1367393

**Published:** 2024-02-20

**Authors:** Raffaele Palmieri, Atto Billio, Felicetto Ferrara, Sara Galimberti, Roberto M. Lemoli, Elisabetta Todisco, Federico Moretti, Adriano Venditti

**Affiliations:** ^1^ Hematology, Department of Biomedicine and Prevention, University Tor Vergata, Rome, Italy; ^2^ Division of Hematology and Bone Marrow Transplant (BMT), Hospital S. Maurizio, Bolzano, Italy; ^3^ Division of Hematology, Cardarelli Hospital, Naples, Italy; ^4^ Hematology Unit, Department of Clinical and Experimental Medicine, University of Pisa, Pisa, Italy; ^5^ Cattedra di Ematologia, Dipartimento di Medicina Interna (DiMI), Università di Genova, Genova, Italy; ^6^ Clinica Ematologica, Istituto di Ricovero e Cura a Carattere Scientifico (IRCCS) Policlinico San Martino, Genova, Italy; ^7^ Struttura Complessa (SC) Ematologia, Ospedale Busto Arsizio, Azienda Socio Sanitaria Territoriale (ASST) Valle Olona, Varese, Italy

**Keywords:** acute myeloid leukemia, clinical prognosticators, disease biology, fitness, acute leukemia

## Abstract

In patients with Acute Myeloid Leukemia (AML), the assessment of disease risk plays a central role in the era of personalized medicine. Indeed, integrating baseline clinical and biological features on a case-by-case basis is not only essential to select which treatment would likely result in a higher probability of achieving complete remission, but also to dynamically customize any subsequent therapeutic intervention. For young high-risk patients with low comorbidities burden and in good general conditions (also called “fit” patients), intensive chemotherapy followed by allogeneic stem cell transplantation still represents the backbone of any therapeutic program. However, with the approval of novel promising agents in both the induction/consolidation and the maintenance setting, the algorithms for the management of AML patients considered eligible for intensive chemotherapy are in constant evolution. In this view, we selected burning issues regarding the identification and management of high-risk AML, aiming to provide practical advice to facilitate their daily clinical management in patients considered eligible for intensive chemotherapy.

## 1 Introduction

Acute myeloid leukemia (AML) refers to a large group of diseases rather than a single malignancy (1). Accordingly, AML classification systems are being constantly updated to better intercept such heterogeneity (2, 3). Both the latest WHO and ICC classifications rely on disease biology at baseline (e.g. genetic/cytogenetics and primary vs. secondary AML) for AML categorization, while neither patient’s clinical features (e.g. age, comorbidities, and performance status) nor disease-related dynamic characteristics (e.g. quality of response and measurable residual disease) are yet firmly incorporated into those models for the definition of disease risk (2, 3). However, in clinical practice, and as recognized by the European Leukemia Net (ELN) recommendations (4), all these aspects need to be considered not only for treatment planning at disease presentation, but also to personalize any subsequent therapeutic intervention (5). This may be particularly relevant for patients with unfavorable clinical and biological characteristics and who are therefore identified as “high-risk”. The treatment of patients belonging to such group has historically been challenging, mainly because of the unsatisfactory results associated with standard chemotherapy delivery. (6). In recent years, after several decades of stagnation, several new drugs are being approved, (7) progressively expanding the therapeutic landscape of high-risk AML. Fueled by these significant advances, we are finally foreseeing a new era in the management of these hard-to-treat diseases. However, as there are still several open issues regarding these complex malignancies, we discussed selected topics concerning the identification and management of high-risk AML, aiming to provide practical advice for the treatment of patients considered eligible to intensive chemotherapy.

## 2 High-risk acute myeloid leukemias: background

### 2.1 Biological factor defining high-risk AML

The definition of high-risk in AML is currently based on clinical and biological characteristics that can be identified either at diagnosis or during the course of the disease (8). From a biological standpoint, selected genetic and molecular abnormalities that can be identified at baseline confer poor response to induction chemotherapy and justify the unsatisfactory long-term outcome observed in this disease category (9–11). Following last years’ major advances in understanding the clinical relevance of specific molecular abnormalities (4), the ELN group has recently released an updated version of recommendations to harmonize diagnosis, response, and prognostic assessment of AMLs. The most relevant changes introduced in this version are represented by the exclusion of the FLT3-ITD allelic ratio in the risk stratification and the clarification of the prognostic relevance of specific genetic/cytogenetic aberrancies (12). Accordingly, AML with FLT3-ITD is now classified as intermediate risk, irrespective of the allelic ratio and *NPM1* mutational status, provided that adverse-risk genetic lesions are absent. Regarding NPM1 mutations, concurrent adverse cytogenetic abnormalities now qualify as adverse risk. Additionally, specific genetic lesions that are predominantly detected in myelodysplastic syndromes (MDS) patients, are now classified as myelodysplasia-related (MR) and included in the adverse-risk list. This adjustment is justified by the evidence that these mutations, typically associated with AML evolving from an antecedent hematologic disease, can also be detected in *de novo* AML and confer adverse risk even in the absence of MR morphological features and/or a prior history of MDS. In line with this, multilineage dysplasia is no longer consider sufficient to define the secondary nature of the disease according to the WHO 2022 classification (2). To a greater extent, the importance of prior medical history (either of MDS, myeloproliferative disorder or of cytotoxic exposure) has been somehow downgraded by both ICC and ELN2022 systems, since such features are currently considered as “diagnostic qualifier” and no longer “disease-defining” of a specific AML-subtype (3, 12). Finally, the adverse-risk group also includes recurring cytogenetic abnormalities, such as t(3q26.2;v) involving the “*MECOM”* gene, or t(8;16)(p11;p13) associated with the “*KAT6A*::*CREBBP”* gene fusion and hyperdiploid karyotypes with multiple trisomies (12), whereas hyperdiploid karyotypes with multiple trisomies are no longer considered as complex karyotypes. The primary clinical utility of the ELN prognostic categorization is represented by the selection of subjects who are candidates for allogeneic stem cell transplantation (alloSCT) in first complete remission (CR), which is the only treatment option that offers a chance of long-term survival to patients with high-risk AML (13).

Together with baseline molecular profiling, additional disease-related features play a central role in defining high-risk AML. Following standard induction chemotherapy, roughly 75-80% of newly diagnosed young adults and 50-60% of older patients with AML are expected to achieve CR or CR with incomplete peripheral blood count recovery (CRi) (14). Refractoriness to anthracyclines plus cytarabine or fludarabine-based regimens after 2 induction cycles converts into a dismal survival irrespective of pretreatment disease biology (15). Furthermore, the quality of response as measured by high-sensitive techniques, such as multiparametric-flow cytometry or sensitive molecular-based approaches, should be also considered for the identification of high-risk patients (16).

### 2.2 Clinical factors influencing the outcome of high-risk AML

Besides disease biology, it is of utmost importance to determine patients’ physical function before initiating any AML treatment. Age has historically been considered a surrogate to determine the eligibility or ineligibility for intensive approaches. In addition, there is robust evidence supporting the role of pre-existing comorbidities in negatively influencing the tolerability to active therapies, although only few studies have investigated this topic prospectively (17, 18). Although both chronologic age and comorbidities may be considered surrogates of functional reserve, their accurateness as single parameters in defining a patient overall “well-being” is limited. Such evaluation appears critical as it represents a first fundamental step in deciding which intensity of treatment would be more appropriate in a specific clinical context. (5) However, although tests for objectively measure physical and also cognitive function are particularly useful, there are no generally accepted criteria to consider a patient eligible/ineligible for active therapy. (12) In daily clinical practice, several scores based on various combinations of age, performance status and comorbidities have been proposed to improve the assessment of patients’ physical condition. All these models (commonly referred to as “fitness scores”) are intended to evaluate the optimal risk/benefit ratio of a given therapy when delivered to a specific patient (5).

Among the available scores for fitness definition, Ferrara et al. proposed a definition of unfitness for intensive and non-intensive chemotherapy that was developed using a Delphi consensus-based process involving a panel of Italian experts belonging to the Italian Society of Hematology (SIE), Italian Society of Experimental Hematology (SIES) and Italian Group for Bone Marrow Transplantation (GITMO) (19). According to the so-called “Ferrara criteria”, patients may be categorized into fit/unfit for intensive chemotherapy, or unfit for non-intensive chemotherapy.

Since their publication, the Ferrara criteria have penetrated the Italian clinical practice and, even though we still lack prospective studies, a strong correlation was shown between short-term mortality and expected outcomes with intensive chemotherapy. In a retrospective analysis of 180 consecutive patients with a median age of 66 years, treated in a single institution (20), a high degree of concordance was observed between fitness categorization according to the “Ferrara criteria” and the treatment intensity that was actually delivered. Consequently, a clear, discrete outcome stratification was observed among fit, unfit and frail patients (median overall survival of 15.3, 8.6 and 1 months, respectively). Consistently, Borlenghi et al. (21) reported on the applicability of the “Ferrara criteria” in a large population of 699 patients treated in 8 Italian hematological institutions. The criteria proved to be easily applicable to 98% of patients and fitness independently predicted survival. The authors concluded that these easy-to-apply criteria, combined with the evaluation of biological factors, could represent a reliable tool to modulate the intensity of available treatments for different AML patients.

The accuracy of the Ferrara criteria was also investigated in a large cohort of 655 adult AML patients receiving intensive chemotherapy in a single US institution. In this study, being categorized as unfit resulted in higher mortality at an early timepoint (mortality was up to 42% in the group of Ferrara unfit patients after 100 days from intensive chemotherapy start) (22).Based on these findings, the Ferrara criteria seem instrumental in facilitating the choice of the most appropriate treatment regimen in AML patients. This tool does not only offer a significant benefit in daily clinical practice but may also allow for greater harmonization of fitness definition in clinical trials. In a future perspective, pre-treatment clinical assessment of adult patients with AML should also include appropriate investigations of physical and emotional functioning. In this view, integrating a complete geriatric assessment may help identify vulnerabilities, otherwise unnoticed or underestimated, that can assist in the decision-making process and supportive management, throughout the entire therapeutic course (23, 24).

**Figure 1:**
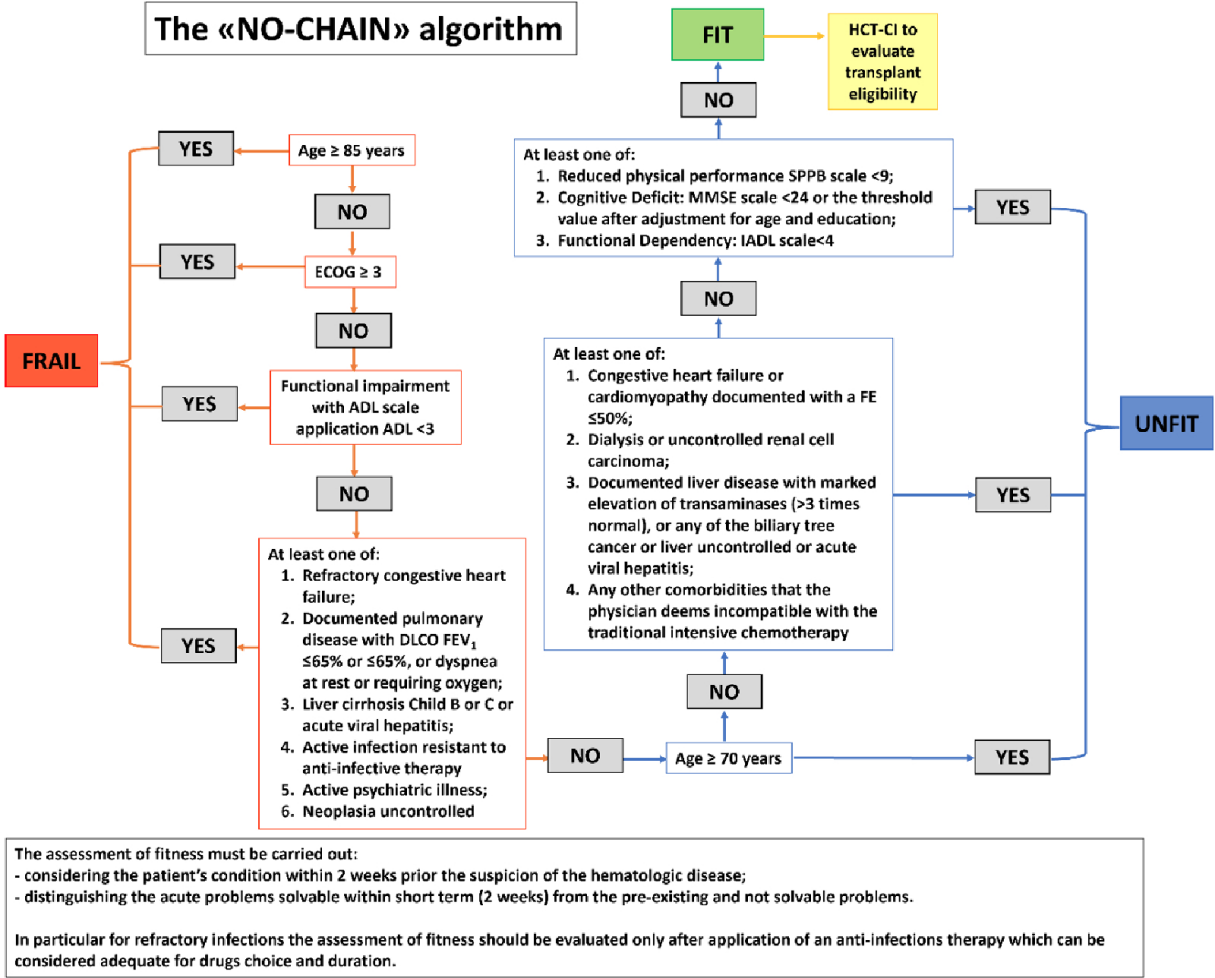
"NO chain" algorithm proposed to assess the various levels of fitness related to intensive chemotherapy in elderly AML patients (>60 years old) (adapted from 25).

An example of an approach intended to facilitate fitness assessment in AML is the “No chain” algorithm displayed in **Figure 1** (25).

In conclusion, the current dynamic prognostic models for AML risk definition should incorporate response to intensive induction therapy, in addition to pretreatment risk factors including genetic and molecular markers as well as age and fitness. The table below summarizes the risk factors that should be taken into account when defining high-risk AML according to the authors (**Table 1**).

**Table 1:** Risk factors defining high-risk AML.

RISK FACTORS	
**Genetic abnormalities** (12)	t(6;9)(p23.3;q34.1)/*DEK*::*NUP214* t(v;11q23.3)/*KMT2A*-rearranged# t(9;22)(q34.1;q11.2)/*BCR*::*ABL1* t(8;16)(p11.2;p13.3)/*KAT6A*::*CREBBP* inv(3)(q21.3q26.2) or t(3;3)(q21.3;q26.2)/*GATA2*, *MECOM(EVI1)* t(3q26.2;v)/*MECOM*(*EVI1*)-rearranged −5 or del(5q); −7; −17/abn(17p) Complex karyotype,** monosomal karyotype†† Mutated *ASXL1, BCOR, EZH2, RUNX1, SF3B1, SRSF2, STAG2, U2AF1, and/or ZRSR2*‡‡ Mutated *TP53* ^a^
**AML sub-type**	Secondary type, after previous hematological diseases or previous cytotoxic therapies (t-AML, AML-MR)
**Clinical factors**	Age Comorbidities Performance Status
**Quality or response**	Refractoriness to anthracyclines and cytarabine or fludarabine-based regimens (2 cycles) MRD positivity after 2 cycles (induction/consolidation)

Excluding KMT2A partial tandem duplication (PTD).

**Complex karyotype: ≥3 unrelated chromosome abnormalities in the absence of other class-defining recurring genetic abnormalities; excludes hyperdiploid karyotypes with three or more trisomies (or polysomies) without structural abnormalities.

††Monosomal karyotype: presence of two or more distinct monosomies (excluding loss of X or Y), or one single autosomal monosomy in combination with at least one structural chromosome abnormality (excluding core-binding factor AML).

‡‡For the time being, these markers should not be used as an adverse prognostic marker if they co-occur with favorable-risk AML subtypes.

^a^TP53 mutation at a variant allele fraction of at least 10%, irrespective of the TP53 allelic status (mono- or biallelic mutation); TP53 mutations are significantly associated with AML with complex and monosomal karyotype.

## 3 Intensive chemotherapy in patients who are not eligible for alloSCT

The successful treatment of high-risk AML patients remains challenging. Indeed, outside clinical trials, only a small proportion of patients (around 40%) aged over 65 years receive intensive chemotherapy to pursue disease eradication (26), and only an ever smaller proportion of them is expected to enter CR without developing severe or even life-threatening complications (22). When it comes to therapy-related (t-AML) and AML with myelodysplasia-related changes (AML-MRC) patients (1), the scenario is further complicated by the presence of all those unique clinical features (such as advanced age, higher comorbidity burden as compared to patients with *de novo* AML, and prior cytotoxic exposure) that preclude the chance for alloSCT delivery in most cases (27).

In patients with AML-MRC and t-AML, treatment with a recently approved liposomal formulation of the “7 + 3” regimen (CPX-351) resulted in a CR/CRi rate of 48% with 31% of patients still alive at 24 months. Even though CPX-351 delivery was associated with prolonged cytopenia and hospitalization, no increased occurrence of early death as compared to the conventional “7 + 3” regimen was observed (30-days mortality: 5.9% *vs* 10.6%; 60-day mortality: 13.7% *vs* 21.2%, for CPX-351 and “7 + 3” respectively) (28).

Moreover, a higher percentage of patients were transplanted when treated with CPX-351 rather than with the “7 + 3” regimen (34% *vs* 25%), translating into a significant prolongation of OS (28). However, even in non-transplanted patients, CPX-351 demonstrated a better OS compared with conventional intensive chemotherapy in terms (14.7 *vs* 7.6 months for CPX-351 vs. "7 + 3”, respectively; HR = 0.57) (29). Such data were confirmed in a 5-year extended follow-up (5-year OS of 18% *vs* 8% for CPX-351 vs.”7 + 3”, respectively); additionally, within the elderly population (between 70 and 75 years old), 16% of patients receiving CPX-351 were still alive at last follow-up compared to none of the patients in the “7 + 3” cohort (7).

The survival advantage of CPX-351 in AML-MRC and t-AML patients was confirmed in real-life settings. In a French study by Chiche et al., among 103 patients treated with CPX-351, CR rate was 55%, with a median OS of 16.1 months. Although alloSCT significantly prolonged OS, even among non-transplanted patients (65% of the whole cohort) median OS was significantly longer than what was observed in a historical cohort of subjects receiving “7 + 3” or demethylating agents. (30).

Such findings were also observed in another cohort of 71 patients receiving CPX-351 in the context of the Italian compassionate use program. CR rate was 70.4% with 37.5% of patients achieving undetectable MRD and 28.2% being transplanted (31). In addition, 70.5% of non-transplanted patients remained alive and progression-free at 12 months, emphasizing the efficacy of CPX-351 also in patients not receiving transplantation (31).

In a German study including 188 patients, 86% and 14% of patients received one and two induction cycles of CPX-351 respectively, and 10% received at least one cycle of CPX-351 consolidation. Overall, 22% of patients achieved CR/CRi and 38% of patients did not proceed to alloSCT. In univariate analysis, OS was found to be significantly prolonged by transplant (HR = 5.1), with OS of the whole population reaching a median duration of 21 months (32).

In a real-life study by Matthews et al., the efficacy of CPX-351 was retrospectively evaluated in 217 patients and compared with the combination of venetoclax and azacitidine (ven/aza) delivered to 439 patients. Overall, 72% patients from the CPX-351 cohort and 90% patients from the ven/aza cohort did not undergo alloSCT. Two-year OS was identical (28%) for both groups, while the median OS was 37 months among patients who received alloSCT. In terms of safety, the significantly higher proportion of febrile neutropenia and culture positive infections observed among patients receiving CPX-351 (90% vs 54% [p<0.0005] for febrile neutropenia and 67% vs. 36% [p=0.0004] for patients receiving CPX-351 and ven/aza, respectively), did not translate into an excess of early mortality at 30 and at 60 days (5% vs. 5% at 30 days and 10% vs 13% for CPX-351 and ven/aza, respectively) (33).

A therapeutic program including CPX-351 induction and consolidation proved to be advantageous also in older patients. Among 309 elderly with AML-MRC and t-AML from the Phase III CPX-351 trial, 49 subjects received CPX-351 as consolidation therapy whereas 32 received the “5 + 2” scheme. Receiving CPX-351 was associated with a reduced relapse rate (8% *vs* 20%) and a longer survival (25.4 *vs* 8.5 months) compared to patients treated with “5 + 2” (34).

In conclusion, it appears that for patients with AML-MRC and t-AML (according to WHO 2016 classification) and that are considered eligible to intensive chemotherapy, CPX-351 should be preferred over standard induction chemotherapy. Despite the long-term survival advantage with CPX-351 is more pronounced among patients receiving alloSCT, such benefit was demonstrated also for patients not eligible for alloSCT. This strategy may be taken into consideration for those patients who are considered fit to receive intensive chemotherapy but whose clinical conditions either at baseline or after induction chemotherapy are no (or no longer) permissive for alloSCT.

Regarding patients that are currently classified as AML-MR according to more updated classification systems, we still lack strong evidence on the long-term efficacy of CPX-351 in both transplanted and not transplanted patients.

## 4 Optimal timing for bone marrow evaluation following induction therapy

In patients with AML receiving intensive chemotherapy, the practice of collecting bone marrow after 7-10 days from completion of induction chemotherapy was established to detect situations of “early resistance” and plan appropriate therapeutic interventions in a timely manner. Such a procedure is better known as “day 14 marrow” and is theoretically very attractive since morphologic evaluation of residual leukemic cells is easy to perform and could guide the next therapeutic steps (35). Indeed, previous versions of the NCCN AML guidelines (36) recommended that a “day 14 marrow” aspiration should be performed in order to intercept the morphologic blast persistence as early as possible. Liso et al. investigated the prognostic value of the “day 14 marrow” in 198 *de novo* AML patients, using an age-adjusted blast cut-off of 22% and 15% for patients aged <60 or > 60 years, respectively (37). In this study, among patients who were under the age of 60, CR was achieved in 79% of those with a “day 14 marrow” blast count below 22%. On the other hand, the CR rate was 67% in patients aged 60 years or older whose “day 14 marrow” revealed < 15% blast cell infiltrate. A German study evaluating 449 patients enrolled in the German AML Cooperative Group 1992 trial revealed that bone marrow blast infiltration (assessed on day 16 and analyzed as a continuous variable) correlated to CR rate, OS, and relapse-free survival (RFS) (38). Subsequently, several attempts were made to increase the sensitivity and predictive value of the “day 14 marrow” by reducing the bone marrow blast threshold to 5% (39–42). Among these, a prospective, multicenter analysis of 795 patients showed that patients with a bone marrow infiltration <5% by day 15 had a higher CR rate, higher 5-year event-free survival (EFS), higher RFS and longer OS (42). In the study by Terry et al., a decrease in the blast threshold resulted in a sensitivity ranging from 80% to 90%, meaning that 8-9 patients out of 10 with a “day 14 marrow” blast infiltration lower than 5% were expected to enter CR at the end of induction therapy (43). Even though lowering the “day 14 marrow” blast threshold improved the sensitivity, the specificity of the 5% cut-off is still a matter of debate, and no convincing proofs have demonstrated that this approach reliably reflects the status of morphologic residual disease after induction (43). An additional drawback of this approach is represented by bone marrow hypocellularity, commonly found early after chemotherapy delivery. Indeed, several cooperative groups have demonstrated that the “day 14 marrow” fails to predict CR achievement in 35%-50% of patients, meaning that between 4 and 5 out of 10 patients with bone marrow blast persistence by day 14 can still achieve a CR at the end of induction (44–49). Yanada et al. *evaluated* the correlation between blast count <5% at day 14 and the type of response after induction. The Authors found that only 71.5% of patients achieved a CR at the end of induction (50). Similarly, in a study in which “day-5 marrow” was compared to “day-14 marrow”, blast count below 5% on day 14 predicted CR in 80% of the patients (45). In a retrospective analysis of 194 previously untreated AML patients, Hussein et al. found that “day 14 marrow” was highly sensitive in predicting CR (90% sensitivity) but not OS. Moreover, 43% of the patients with a “day 14 marrow” blast >5% were still able to achieve CR at day 21 or 28 (39). In a retrospective study conducted by Norkin et al., out of 89 patients with a “day 14 marrow” blast count >5% who did not receive a second induction, 32 (36%) subsequently reached CR upon hematologic recovery. If a second course of induction had been automatically given to patients with residual disease according to the “day 14 marrow” assessment, 35%–50% of them would have been over-treated (48).

The “day-14 marrow” strategy of response assessment poses serious safety and economic considerations. Therefore, the decision to give a re-induction based on the “day 14 marrow” assessment should be carefully evaluated, since a second course of induction delivered before full hematopoietic recovery increases the risk of prolonged myelosuppression and life-threatening complications. It has been well documented that treatment-related mortality is significantly higher following a re-induction compared with the first cycle (6.8% *vs* 1.8% respectively) (43). Patients who are given a second cycle of induction therapy also impose a higher economic burden due to the increased use of transfusion support, antibiotics and prolonged hospital staying. Based on these data, the most recent versions of the AML guidelines have somehow downgraded the importance of the “day 14 marrow” assessment (12, 51, 52). The current guidelines suggest that if a bone marrow aspiration is performed between days 14-21 from induction to demonstrate aplasia or blast persistence, a second collection should be performed upon full hematologic recovery to demonstrate CR. In an era in which new agents, such as CPX-351, are available and show considerable myelosuppressive effects and prolonged pancytopenia, the recommendation to defer the “day 14 marrow” assessment is even stronger (28, 31).

In conclusion, the Authors agree that the most informative timepoint to evaluate response in clinical practice should generally remain between days 21-28 in patients receiving standard induction therapies (such as “7 + 3”), or later in patients treated with CPX-351, at time of hematopoietic recovery.

## 5 Consolidation chemotherapy post-induction – for who and how?

Several investigations have addressed the role of post-remission consolidation therapy for younger (under the age of 60) AML patients. The Cancer and Leukemia Group B (CALGB), (53), randomized AML patients of all ages to receive 4 courses of cytarabine consolidation at three different schedules: every 12 hours on day 1, 3, and 5, or as continuous intravenous (IV) infusion at 0.4 g/m^2^ or 0.1 g/m^2^ for five days. The results showed improvements in OS and DFS for patients under 60 years old receiving high-dose cytarabine (3 g/m^2^). Based on these findings, high-dose cytarabine-based consolidation has become the standard of care for patients with AML after achieving remission with standard-induction therapy (53). The influence of different doses of cytarabine on clinical outcomes according to cytogenetics was further explored in a subsequent prospective CALGB study (54). A total of 285 AML patients were enrolled and stratified into 3 groups: core binding factors (CBF) leukemias, normal karyotype, and patients with other cytogenetic and molecular abnormalities. The data demonstrated a significant impact of high-dose cytarabine on long-term remission in the CBF and normal karyotype AML groups, whereas patients with other cytogenetic alterations showed an uncertain outcome, irrespective of cytarabine dose and schedule (54).

The Medical Research Council (MRC) AML 15 trial investigated the optimal consolidation cytarabine dose by randomizing AML patients under the age of 60 to receive 3 g/m^2^ *vs* 1.5 g/m^2^. Although the results showed a trend towards a lower relapse rate for patients who received the higher dose of cytarabine, OS was not significantly different. Moreover, additional supportive care and longer hospitalization were required in the group receiving 3 g/m^2^ (55).

More recently, Magina et al. (56) carried out a systematic review and meta-analysis of several studies evaluating a total of 8877 AML patients of any age and comparing different doses of cytarabine as consolidation. Of note, the authors focused on the cumulative dose because both the number of cycles and dose of single cytarabine infusions varied across studies. A threshold of 20 g/m^2^ was set to discriminate between high-dose *vs* intermediate/low-dose. No advantage of any combination chemotherapy over cytarabine monotherapy was observed in terms of RFS or OS. Similarly, no differences in RFS and OS were observed between the two groups receiving cytarabine at high *vs* intermediate/low doses, even in young (< 64 years) patients. However, high-dose cytarabine provided a statistically significant RFS advantage for the cytogenetic favorable-risk group, along with a non-statistically significant benefit in OS (56).

In conclusion, despite high doses of cytarabine are usually preferred in consolidation, the optimal dose to be used remains to be established in both young and elderly patients.

The optimal number of post-remission chemotherapy cycles was investigated in the MRC AML17 trial enrolling patients who were not considered at high risk according to a comprehensive evaluation of age, blast count, primary vs. secondary disease and cytogenetics (57). To date, no studies have been conducted to evaluate the most effective number of cycles to be adopted in high-risk patients. In addition, it should be underlined that alloSCT in first CR is still considered the treatment of choice for fit, high-risk AML patients (58).

Regarding older patients, we still lack strong evidence on the role of different doses and number of cytarabine consolidation courses. According to results obtained by Mayer et al., patients older than 60 years were not characterized by a higher probability of remaining disease-free after receiving a high-dose cytarabine consolidation (53), even though post-remission therapy has improved the survival of some patients in remission after standard induction (59). Therefore, balancing the risk of toxicity with the possible benefit of improving OS is crucial when considering consolidation therapy in older patients.

In the context of patients not receiving “7 + 3” or other conventional induction regimens, the role of consolidation therapy is still under investigation. In this view, Kolitz et al. analyzed the survival of high-risk AML patients (age between 60 and 75 years, AML-MRC and t-AML) who received CPX-351 or conventional “7 + 3” and that, once in CR, were treated with up to two additional CPX-351 consolidation cycles (34). Although we lack evidence comparing the cytarabine dose contained in the liposome of CPX-351 to the total dose of cytarabine that is actually delivered as monotherapy in the consolidation setting, the subsequent exploratory analysis of this trial showed a statistically significant improvement in OS for patients treated with CPX-351 (7).

In conclusion, the Authors suggest the administration of up to 2 consolidation cycles with an intermediate dose of cytarabine after induction therapy in patients in CR who are not eligible for transplantation. Similarly, the Authors also recommend up to two consolidation cycles with CPX-351 in patients with AML-MRC and t-AML in CR after induction chemotherapy, that are not eligible for alloSCT. Conversely, AlloSCT should be the treatment of choice in high-risk patients who are considered fit enough to receive transplant. In these cases, transplant should be delivered as soon CR is achieved and a suitable donor is identified.

## 6 Outpatient consolidation chemotherapy

Intensive treatment of AML generally consists of induction chemotherapy followed by a variable number of consolidation cycles. Each cycle is associated with 2–3 weeks of severe pancytopenia during which patients require transfusion support and are susceptible to infections. Traditionally, intensive AML treatments have been administered on an inpatient basis due in part to chemotherapy regimen infusion requirements and the need for transfusion support, but also to allow for close monitoring for infectious and other complications. However, outpatient programs for high-dose chemotherapy followed by allogeneic or autologous hematopoietic stem cell rescue represent a viable option in different hematologic diseases (60–68). Over the last years, crucial improvement in supportive care delivery and optimization of antibiotics use, together with the approval of new agents that may not require IV continuous infusion (such as CPX-351), are changing the paradigm for which intensive chemo “must” be administered as inpatient (69–71).

Allan et al. (72) conducted a review of consecutive patients with AML receiving intensive chemotherapy between January 1996 and July 1998, to evaluate the safety and feasibility of early outpatient supportive care. A total of 10 out of 19 patients treated with induction chemotherapy (53%) were discharged within 10 days from treatment start (median 4.5 days). A median of 1.5 readmissions was observed, even though the median hospital stay and in-hospital antibiotic use were both significantly reduced as compared to patients who spent the whole aplastic phase as inpatients (decreased by 30% and 57%, respectively). No significant differences in transfusion requirements or episodes of febrile neutropenia were noticed between the two groups. The 18 patients who survived induction received a total of 31 cycles of consolidation therapy. Early hospital discharge (EHD) was feasible in 30 out of the total 31 cycles (97%) (72).

Moller et al. (73) conducted a prospective study enrolling 60 patients with acute leukemia (54 with AML) receiving outpatient follow-up after EHD. After induction chemotherapy, EHD was feasible in 48 out of 73 (65.7%) patients, with no readmission in 19 cases (40%). No readmission occurred during 69/116 (59%) subsequent cycles of consolidation therapy that benefited from EHD. Neutropenic fever was the prevalent cause of readmission, but no treatment-related deaths were registered (73). In a retrospective study by Eisele et al. (74), 103 consolidation cycles were evaluated. Patients received inpatient treatment and were discharged if they showed satisfactory clinical condition. Patients were discharged after a median of 7 days in 95/103 cycles (92%). Patients were electively readmitted in 45 cycles to initiate chemotherapy-induced cytopenia after a median of 12 (9-16) days whereas outpatient care was the strategy of choice in 45 cycles. Rehospitalization was necessary in 23/50 outpatient cycles (46%), mainly because of neutropenic fever. One patient died during outpatient observation, accounting for an overall mortality of 2% (74).

A total of 178 adults receiving intensive induction chemotherapy were examined in the largest prospective study evaluating EHD (75). After completion of chemotherapy, 107 patients satisfied the pre-designated medical and logistical criteria for EHD, while 29 patients met medical criteria only and served as inpatient controls. EHD patients received outpatient supportive care until count recovery (median 21 days, range 2–45 days). Four patients were discharged early (4%) and none of the inpatient controls died within 30 days from enrollment. Nine patients were discharged early (8%) and none of the inpatient controls required intensive care unit-level care. The average number of red blood cell or platelet transfusions was not statistically different. EHD patients were characterized by a significantly higher number of positive blood cultures but required fewer days of IV antibiotics (75).

More recently, Girmenia et al. prospectively evaluated the feasibility and safety, as well as clinical characteristics, of outpatient AML management during the post-induction phase. In this study, patients receiving consolidation therapy as inpatients were discharged after treatment delivery. A total of 111 patients and 133 consolidation courses were included in the final analysis. Rehospitalizations occurred in 69 cases (54%), mainly because of fever; one patient died due to brain hemorrhage (76).

Outpatient administration of CPX-351 in AML-MRC and t-AML patients has been evaluated in both the consolidation and induction settings with results that suggest the feasibility and safety of this approach in selected patients. In the randomized phase 2 clinical trial that led to the approval of CPX-351, consolidation was administered as outpatient in 40.5% of the cases. CPX-351 improved the duration and proportion of time spent as an outpatient as compared to 7 + 3 with a similar safety profile. Among responding patients, the number of days in hospital for induction plus consolidation was similar to 3 + 7 (median 42 *vs* 43 days) with fewer days of hospitalization required for consolidation in the CPX-351 arm (median 4 *vs* 11 days) (77).

In a subsequent exploratory analysis of the pivotal Phase III trial, among patients receiving CPX-351 consolidation, CPX-351 was administered entirely as outpatient in 51% of the cases during the first consolidation cycle, and in 61% during the second cycle. In line with what was observed in the phase 2 trial, no impact of such approach was reported in terms of safety and OS (34).

**Figure 2:**
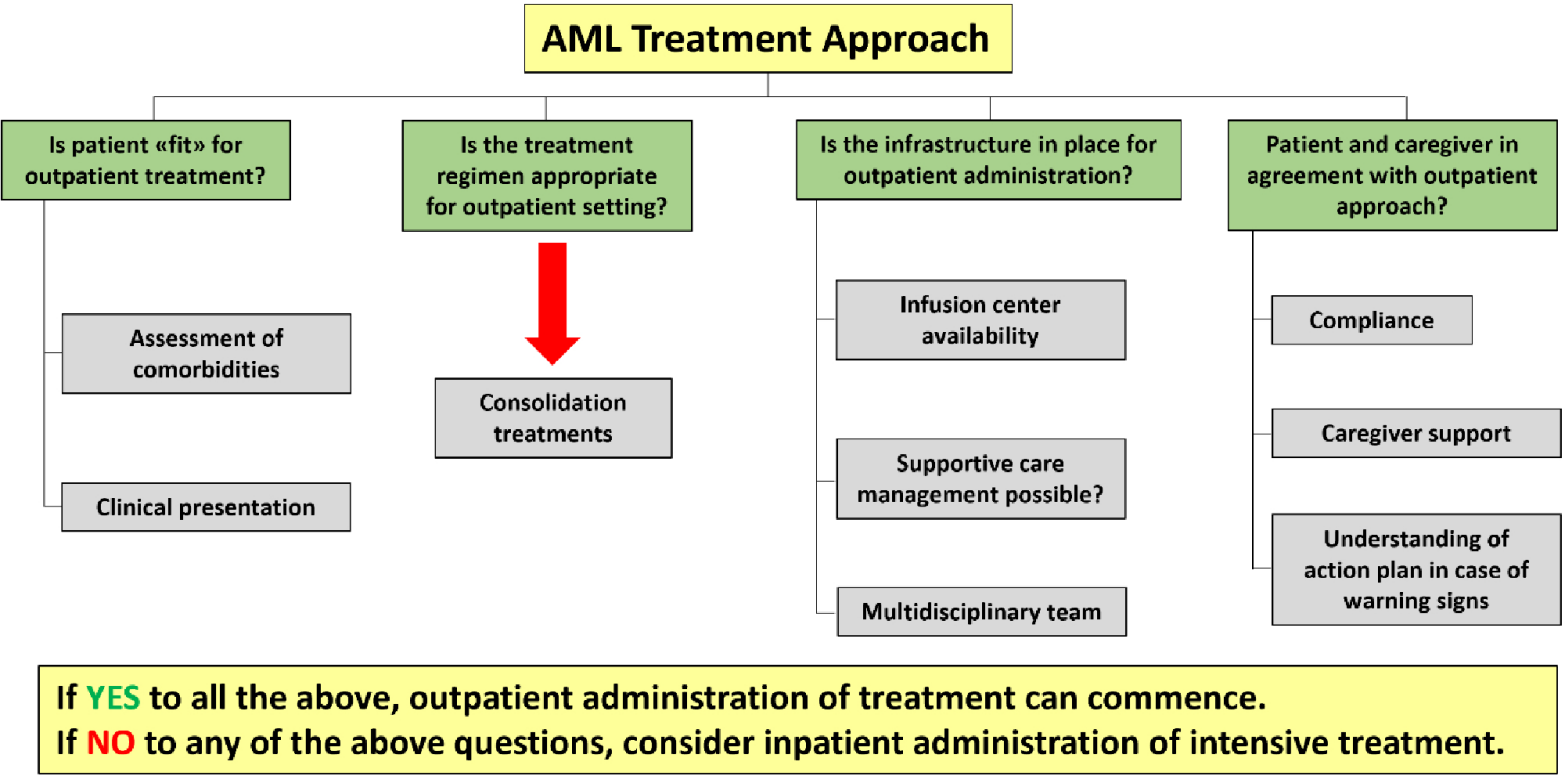
Roadmap of AML outpatient treatment approach (adapted from [81]).

In summary, several studies suggest the feasibility and the cost-effectiveness of outpatient intensive chemotherapy administration. (78–80) Potential benefits include, beside a remarkable reduction of healthcare costs, a decreased risk of hospital-acquired infections. An additional benefit may be a substantial improvement in patients’ quality of life, since hospital stay would be limited to the administration of chemotherapy and to the time strictly necessary to manage side effects (if any). However, EHD can be considered safe if careful planning and close patients monitoring can be ensured. (78–80) Patients’ education is also necessary to report any possible serious complication in a timely manner (78);. If not adequately reported and managed, side effects occurring during chemotherapy-induced aplasia may be rapidly progressing and lead to treatment-related mortality. Accordingly, EHD strategy seems worthy of consideration in the management of selected AML patients, as suggested in **Table 2** and **Figure 2**.

**Table 2:** Factors supporting early hospital discharge of AML patients and related barriers [adapted from (81)].

Summary of factors supporting early hospital discharge and barrier to implementation in patients with acute myeloid leukemia	
**Factors supporting EHD**	**Barrier to implementation**
High costs of inpatient AML care	Geographic logistics; housing, weather
Impaired quality of life in AML related to time spent hospitalized	Infusion center with limitation in space/hours
Limited bed availability necessitating need to discharge patients	Limited bed availability for readmission
New drug approvals for AML	Lack of patient/hospital resources
Improvements in supportive care	Highly comorbid patient population
Decreasing treatment-related mortality in AML over time	Lack of caregivers available
Retrospective and prospective data supporting safety/feasibility of EHD	High risk of infection

AML, acute myeloid leukemia; EHD, early hospital discharge.

In conclusion, the Authors believe that high-dose cytarabine consolidation may be safely managed with an early discharge strategy in selected patients. Similarly, CPX 351 outpatient consolidation may be administered when appropriate patient and logistic factors are fulfilled, and multidisciplinary team support and close monitoring are available.

## 7 Maintenance therapy - for who and how?

Historically, the standard of care for patients with AML achieving CR after induction and consolidation and ineligible for allotransplantation consisted of observation without maintenance therapy (82). Several clinical trials evaluated the role of cytotoxic chemotherapy or even immunotherapy in the maintenance setting but failed to show consistent benefits in OS, while occasionally showed a benefit in RFS (83–86).

Nevertheless, given the high rate of relapse in patients with high-risk AML (87), more treatment approaches are currently being evaluated as maintenance therapy. Over the last decade, the immune checkpoint inhibitors (anti-PD-1, PDL1, and CTLA-4), alone or combined with hypometylating agents (HMA), showed preliminary promising results in patients considered at high risk of relapse (such as MRD-positive ones) (88, 89). In recent years, encouraging results were reported with the oral formulation of azacitidine in the QUAZAR AML-001 trial (90). This study enrolled patients aged 55 years or older who were within 4 months of achieving first CR or CRi after intensive induction chemotherapy and were not candidates for alloSCT. Oral azacitidine was administered at the dose of 300 mg daily on days 1–14 of 28-day cycles until disease progression or unacceptable toxicity. A total of 472 patients underwent randomization; 238 were assigned to receive CC-486 and 234 were assigned to the placebo group. Median OS was significantly longer with CC-486 than placebo (24.7 months and 14.8 months, respectively; p<0.001). Median RFS was also significantly longer with CC-486 as compared to placebo (10.2 months and 4.8 months, respectively; p<0.001). The benefits of CC-486 with respect to OS and RFS were shown in most subgroups, especially in patients ≥65 years old, patients in CR, and patients that were MRD negative at study inclusion. The most common adverse events in both groups were grade 1 or 2 gastrointestinal events. Common grade 3 or 4 adverse events were neutropenia (in 41% of patients in the CC-486 group and 24% of patients in the placebo group) and thrombocytopenia (in 22% and 21%, respectively). Overall, health-related quality of life was preserved during CC-486 treatment (90).

Multiple targeted therapies are being tested in the maintenance setting in AML with specific molecular alterations. Incorporation of maintenance therapy by delivering the oral formulation of a given targeted therapy until relapse or unacceptable toxicity, represents an attractive strategy. Several studies have investigated this approach in FLT3-mutated patients, including the RATIFY (midostaurin + chemotherapy +/-alloSCT) (91), SORAML (sorafenib + chemotherapy+/- alloSCT (92), and QuANTUM-First phase 3 study (93). The latter showed that the addition of quizartinib to standard chemotherapy with or without alloSCT followed by maintenance therapy for up to 3 years resulted in improved OS in adults aged 18–75 years with FLT3-ITD-positive AML (93). Based on the results from the QuANTUM-First trial, quizartinib provides a new, effective, and generally well-tolerated treatment option for adult patients with previously untreated FLT3-ITD-positive AML. The feasibility of maintenance therapy is being investigated among IDH1/2 mutated patients with promising results (94, 95).

For patients receiving alloSCT, post-transplant maintenance has the potential to prolong long-term both overall and disease survival. Such strategy may be advantageous in those patients with high-risk of post-transplant relapse, including those with an MRD positive status. Multiple studies have been conducted regarding the use of a myriad of agents in the post-alloSCT maintenance setting, in some cases with promising results (96). In a retrospective study from Ali et al., post-alloSCT azacitine maintenance therapy improved EFS and OS in patients receiving reduced-intensity transplant (97). Based on these findings, azacytidine in combination with the BCL-2 inhibitor venetoclax is also currently under investigation as maintenance therapy in patients with MRD after alloSCT (NCT04128501). Regarding the use of FLT3 inhibitors after alloSCT, sorafenib monotherapy showed to be effective in reducing the risk of relapse in a multicenter, randomized, phase 3 trial (cumulative 1-year incidence of relapse of 7.0% vs. 25% for sorafenib and placebo, respectively; p=0.0010) (98). An additional potential strategy to reduce the risk of post-transplant relapse is represented by donor lymphocyte infusion (DLI). DLI is a kind of immunotherapy that has shown to be effective to reduce the risk of relapse by enhancing the graft-versus-leukemia (GVL) effect. As a major drawback, DLI is associated with high risk of severe GVHD and, in turn, of GVHD-induced mortality (99).

In light of the above evidence, the Authors suggest administering maintenance treatment with oral azacytidine for adult patients in CR after intensive induction and consolidation chemotherapy and not candidates for alloSCT. For patients receiving alloSCT, further investigation is needed to support the systematic use of any maintenance strategy after transplant.

## 8 Conclusions

After nearly four decades characterized by a lack of novelties, we are finally entering a new era in the management of high-risk leukemias. Compared to the past, identifying patients from such group is no longer limited to descriptive purposes but extends to practical implications. The constant updating of disease-risk stratification systems, together with the refinement of the available tools for fitness assessment, are progressively improving our ability to estimate the risk/benefit ratio of a given therapy on a case-by-case basis. Accordingly, identifying all the clinical (e.g. patient-related) and the biological (e.g. disease-related) features that may negatively influence the success of the therapeutic program is mandatory in order to offer the “best” treatment option to each patient. For high-risk patients considered eligible for intensive approaches, standard induction chemotherapy followed by alloSCT has historically represented the strategy of choice. In recent years, new-generation drugs have shown to be more effective and better tolerated than conventional cytotoxic agents in specific settings. As such agents were also associated with discrete outcomes in patients not receiving alloSCT, we may speculate that a possible revolution on what we currently define as “standard of care” may finally be around the corner. Additionally, introducing selected drugs in the maintenance setting has somewhat softened the line between intensive and non-intensive approaches, underlying the importance of re-evaluating both patient and disease status during the course of the disease. As a possible drawback, additional investigation is still needed to better understand practical aspects related to the use of these novel agents (such as timing and frequency for response assessment). In a future perspective, extrapolating data from real-world studies is expected to shed light on the efficacy and tolerability of newly approved drugs, with the final aim of further improving the management of high-risk leukemias **Table 3**.

**Table 3:** Summary of authors’ practical consideration regarding the management of high-risk AML.

Topic	Practical consideration
**Role of intensive chemotherapy in patients who are no eligible to alloSCT**	Since standard induction and consolidation chemotherapy have been associated with prolonged complete remission even in patients not submitted to transplant, it should be taken into consideration irrespective of alloSCT eligibility.
**Optimal timing for bone marrow evaluation following induction therapy**	The most informative timepoint to evaluate response in clinical practice should be between day 21-28 in patients receiving standard induction therapies (such as “7 + 3”), or later in patients treated with CPX-351.
**Role of consolidation chemotherapy after CR achievement**	Administering up to 2 consolidation cycles with intermediate dose of cytarabine after induction therapy should be the strategy of choice in patients in CR who are not eligible to transplantation. The authors also recommend up to two consolidation cycles with CPX-351 in patients with AML-MRC and t-AML in CR after induction chemotherapy.
**Outpatient consolidation chemotherapy**	With several studies suggesting the feasibility and safety, as well as the cost-effectiveness of outpatient intensive AML consolidation, this strategy seems worthy of consideration in patients meeting selected medical and logistical pre-requisites.
**Indications for maintenance therapy in patients in CR after induction chemotherapy**	Maintenance treatment with oral azacytidine can be administered in adult patients in CR after intensive induction and consolidation chemotherapy and not candidates for alloSCT.

alloSCT, allogeneic stem cell transplantation; CR, complete remission; AML-MRC, acute myeloid leukemia with myelodysplastic-related changes; t-AML, therapy-related acute myeloid leukemia.

## Author contributions

RP: Conceptualization, Writing – original draft, Writing – review & editing. AB: Writing – original draft, Writing – review & editing. FF: Writing – original draft, Writing – review & editing. SG: Writing – original draft, Writing – review & editing. RL: Writing – original draft, Writing – review & editing. ET: Writing – original draft, Writing – review & editing. FM: Writing – original draft, Writing – review & editing. AV: Supervision, Writing – original draft, Writing – review & editing.
